# Human TRMT112-Methyltransferase Network Consists of Seven Partners Interacting with a Common Co-Factor

**DOI:** 10.3390/ijms222413593

**Published:** 2021-12-18

**Authors:** Baiba Brūmele, Margit Mutso, Lilian Telanne, Kadri Õunap, Karīna Spunde, Aare Abroi, Reet Kurg

**Affiliations:** Institute of Technology, University of Tartu, 50411 Tartu, Estonia; baiba.brumele@ut.ee (B.B.); margit.mutso@ut.ee (M.M.); lilian.telanne@gmail.com (L.T.); ounap.kadri@gmail.com (K.Õ.); spunde.carina@gmail.com (K.S.); aare.abroi@ut.ee (A.A.)

**Keywords:** methyltransferase, TRMT112, RNA methylation, ribosome biogenesis, protein stabilisation

## Abstract

Methylation is an essential epigenetic modification mainly catalysed by S-Adenosyl methionine-dependent methyltransferases (MTases). Several MTases require a cofactor for their metabolic stability and enzymatic activity. TRMT112 is a small evolutionary conserved protein that acts as a co-factor and activator for different MTases involved in rRNA, tRNA and protein methylation. Using a SILAC screen, we pulled down seven methyltransferases—N6AMT1, WBSCR22, METTL5, ALKBH8, THUMPD2, THUMPD3 and TRMT11—as interaction partners of TRMT112. We showed that TRMT112 stabilises all seven MTases in cells. TRMT112 and MTases exhibit a strong mutual feedback loop when expressed together in cells. TRMT112 interacts with its partners in a similar way; however, single amino acid mutations on the surface of TRMT112 reveal several differences as well. In summary, mammalian TRMT112 can be considered as a central “hub” protein that regulates the activity of at least seven methyltransferases.

## 1. Introduction

Methylation is an essential epigenetic modification that occurs on a wide variety of substrates in cells. Methylation is mainly catalysed by S-Adenosyl-L-methionine (AdoMet)-dependent methyltransferases (MTases) that are known to transfer methyl groups to nucleic acids, proteins, carbohydrates, lipids and small molecules [1,2]. A remarkable diversity of substrates also require a variety of different enzymes for their modification. A significant percentage of MTases in higher eukaryotes are devoted to the methylation of the translational machinery. This occurs extensively in ribosomal, transfer and messenger RNAs, ribosomal proteins and translation factors, and is important for the structure stability, ribosome biogenesis and translation fidelity [3,4,5].

TRMT112 is a small evolutionarily conserved protein that acts as a co-factor and activator for different MTases involved in rRNA, tRNA and protein methylation [6,7,8,9,10,11,12]. The most characterised member of the family is Trm112 from yeast *Saccharomyces cerevisiae*. Many works have shown that the deletion of the *Trm112* gene leads to a slower growth of yeast [13,14,15]. Studies on SMO2, the *Arabidopsis thaliana* Trm112 orthologue, have also demonstrated that an inactivation of the SMO2 gene leads to a defect in cell growth and causes disorders in cell division and development [16].

The TRMT112 protein forms heterodimers with several partners in mammalian cells. The TRMT112 interaction with 18S rRNA MTases WBSCR22 (BUD23, MERM1) and METTL5 [9,10,12,17] is required for their metabolic stability and enzymatic activity in cells. WBSCR22, mediating the N7-methylation of G1639 in 18S rRNA, is involved in pre-rRNA processing and ribosome small subunit biogenesis, and the knock-down of WBSCR22 results in a slower growth of cells [18]. The homozygous deletion of *Bud23* results in embryo-lethality in mice [19]. However, the methylation activity of WBSCR22 is not required for ribosome biogenesis [12], suggesting that the protein itself, rather than its enzymatic activity, is important for the formation of small ribosomal subunits. The other rRNA MTase METTL5, responsible for decorating the 18S rRNA with one m^6^A, has no significant impact on cell growth and rRNA maturation [10]. The TRMT112 protein forms a stable complex with protein MTase N6AMT1 (HEMK2), catalysing the N5-methylation of the glutamine residue in the GGQ motif of eukaryotic translation termination factor 1 (ETF1) [20]. In addition, it possesses histone lysine MTase activity, writing the monomethylated chromatin mark on H4K12 in vitro and in vivo. The methylation of H4 by N6AMT1 controls the expression of genes encoding proteins involved in the cell cycle [8]. For protein methylation activity, N6AMT1 requires TRMT112 as a partner; N6AMT1 alone is not sufficient [8,21]. Recently, N6AMT1 was described as a DNA N6-adenine MTase, which functions as a monomeric protein [22]. However, structural analysis shows that the surface surrounding the active site in N6AMT1 is largely negatively charged, which is unsuitable for the binding of a nucleotide substrate [21], suggesting that N6AMT1 is a bona fide protein rather than a DNA MTase. This was confirmed later by a comparative in vitro analysis using a N6AMT1(HEMK2)-TRMT112 heterodimer purified from *E.coli* [23]. The loss of N6amt1 leads to early embryonic lethality in mice. The post-implantation development of mutant embryos is impaired, resulting in degeneration around embryonic day 6.5 [24]. In contrast to WBSCR22 and METTL5, the metabolic stability of N6AMT1 is not impaired by the downregulation of TRMT112; however, the expression of N6AMT1 isoforms in cells is regulated through the interaction with TRMT112 [25]. The TRMT112 protein forms a heterodimer with ALKBH8, a tRNA-hypermodification enzyme that is required for DNA damage survival [26,27]. In addition to the MTase domain, which catalyses the methylation of cm5U to mcm5U in several tRNAs, ALKBH8 also has an oxygenase domain catalysing hydroxylation of wobble 5-methoxycarbonylmethyluridine, whereas both enzymatic activities can work independently [28]. THUMPD3, together with TRMT112, is a m^2^G methyltransferase working on a broad range of tRNA substrates. THUMPD3-knockout cells exhibit an impaired global protein synthesis and reduced growth [29].

The crystal structures of human TRMT112 and its orthologues in yeast, together with N6AMT1/Mtq2, Bud23, METTL5 and Trm11 [7,8,10,11,21,30,31,32], have revealed that TRMT112 interacts with its partners in a similar way. TRMT112 covers a large hydrophobic cluster of the partner protein and partially protects it from the external environment, thereby increasing its solubility and stability. In addition, its interaction with TRMT112 enhances the binding of SAM (S-adenosyl methionine) to the TRMT112-MTase complex [8,30]. All MTases use the same surface of their MTase domain to bind to the same region of TRMT112, raising the question of whether they compete to interact with TRMT112.

To uncover the TRMT112-MTase network in mammalian cells, we performed a SILAC (stable isotope labeling by amino acids in cell culture) assay to pull-down all MTases interacting with the common co-activator TRMT112. Our data show that the human TRMT112 interacts with at least seven different MTases in mammalian cells. The TRMT112 level and localisation in the cells are tightly regulated, and there is a mutual feedback loop between TRMT112 and its partners. TRMT112 interacts with its partners in a similar way, but single amino acid mutations on the surface of TRMT112 reveal several differences as well. In summary, mammalian TRMT112 can be considered as a central “hub” protein that regulates the activity of several MTases.

## 2. Results

### 2.1. Identification of TRMT112 Interaction Partners

**Figure 1:**
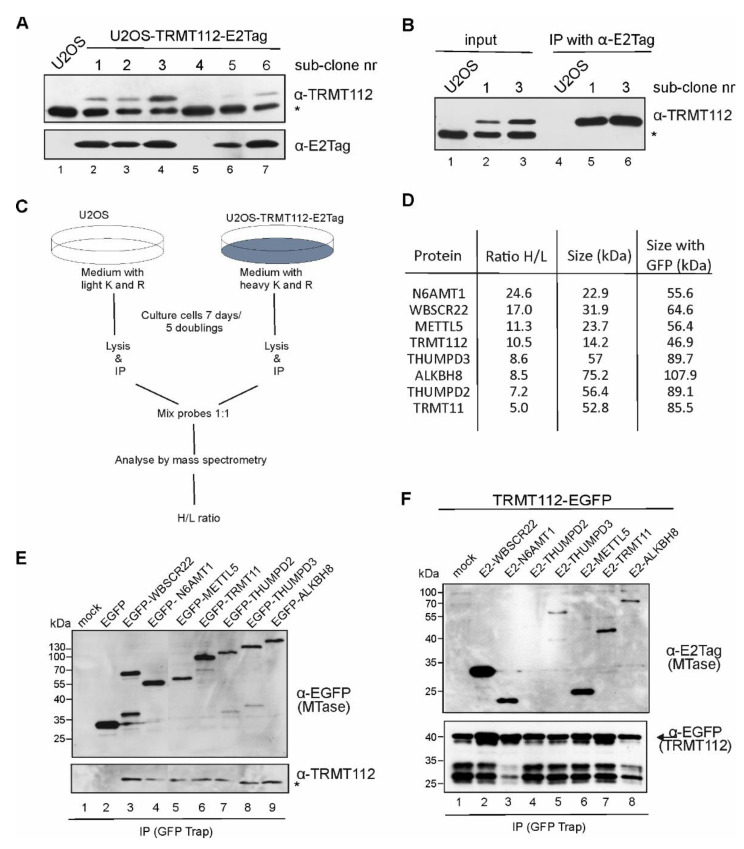
TRMT112 interacts with seven MTases in mammalian cells. (**A**) Generation of stable U2OS cell lines expressing the TRMT112-E2Tag protein. Protein expression was detected by Western blot analysis using antibodies against TRMT112 and E2Tag epitope tag. Images of results with anti-TRMT112 depict both recombinant (upper band) and endogenous protein (lower band marked with asterisk *). (**B**) Immunoprecipitation analysis of TRMT112 from stable cell lines using E2Tag antibodies. (**C**) Experimental scheme of the SILAC assay coupled co-immunoprecipitation assay. (**D**) MTases interacting with TRMT112 pulled down in SILAC screen. H/L ratio shows the relative enrichment of proteins pulled down with TRMT112 compared to mock control. (**E**) The endogenous TRMT112 protein interacts with all seven MTases identified with SILAC assay. EGFP-fused MTases were expressed in U2OS cells and immunoprecipitated using GFP-Trap beads followed by Western blot analysis with EGFP and TRMT112 antibodies. (**F**) TRMT112-EGFP fusion protein was expressed together with epitope tagged-MTases in U2OS cells, and GFP-Trap beads were used to immunoprecipitate TRMT112-EGFP protein. Western blot analysis was performed with antibodies against E2Tag to detect MTases, and EGFP for analysing TRMT112-EGFP protein levels in immunoprecipitated probes.

To identify the TRMT112-protein-interacting partners in mammalian cells, we performed a SILAC assay coupled with a co-immunoprecipitation assay similar to our previous study [9]. First, the stable TRMT112-E2Tag expressing U2OS cell line was generated. The epitope tag E2Tag was inserted into the C-terminus of the protein, as our previous studies have shown that the N-terminal epitope tag interrupted the interaction surface of TRMT112 and WBSCR22 [9]. An immunoblot analysis of six different subclones confirmed that TRMT112-E2Tag is expressed in most of the stable cell line clones obtained, though at different levels (Figure 1A). We noticed that the expression of recombinant TRMT112 had a decreasing effect on the endogenous TRMT112 protein levels (Figure 1A upper panel, the star refers to the endogenous protein). The recombinant TRMT112 protein was immunoprecipitated from the cell lysates of stable cell line clones using antibodies against E2Tag (Figure 1B), confirming that these cells could be used for further analysis.

The scheme of our SILAC experiment is shown in Figure 1C. For the SILAC analysis, the subclone 3 (Figure 1B) was used. We performed three biological replicates and, in all cases, seven MTases with H/L ratios ranging from 5 to 24 were pulled down (Figure 1D). Among these proteins were the already known TRMT112 interaction partners WBSCR22 [9,13], N6AMT1 [20], ALKBH8 [26], METTL5 [10] and THUMPD3 [29], but also novel, uncharacterised proteins THUMPD2 and TRMT11. THUMPD2 is specific to mammalian cells, but TRMT11 homologue in yeast Trm11 is a tRNA MTase [30]. According to the molecular function, most of the TRMT112 interaction partners belong to the term “RNA MTase activity” (GO:0008173), annotated either by experimental data (METTL5, WBCSR22, ALKBH8 and THUMPD3) or by in silico prediction (TRMT11 and THUMPD2) [33,34].

To confirm the proteomics results, we constructed EGFP-fusion proteins with all identified MTases and performed an immunoprecipitation analysis using the GFP-Trap system. As shown in Figure 1E, all EGFP-fusion proteins were expressed in U2OS cells with expected sizes and were able to immunoprecipitate the endogenous TRMT112 protein from the cell lysate (Figure 1E, lower panel). In addition, we constructed TRMT112-EGFP fusion, where EGFP was placed in the C-terminus of TRMT112. Unfortunately, TRMT112-EGFP fusion was less stable than others and resulted in degradation products, as shown in Figure 1F lower panel (the arrow refers to the correct fusion protein, with size 46 kDa). Despite this, we were able to immunoprecipitate all recombinant MTases from the cells electroporated with two expression constructs, one coding for TRMT112-EGFP and the other E2 epitope tagged-MTase (Figure 1F, upper panel). The strongest signal was observed with WBSCR22, followed by N6AMT1 and METTL5 (Figure 1F, lanes 2, 3, 6), which also showed the highest H/L ratio (Figure 1D) in the SILAC assay. The weakest, almost undetectable signal in this co-expression assay was with THUMPD2 (lane 4, Figure 1F).

### 2.2. TRMT112-Interacting MTases Co-Localise with TRMT112

**Figure 2:**
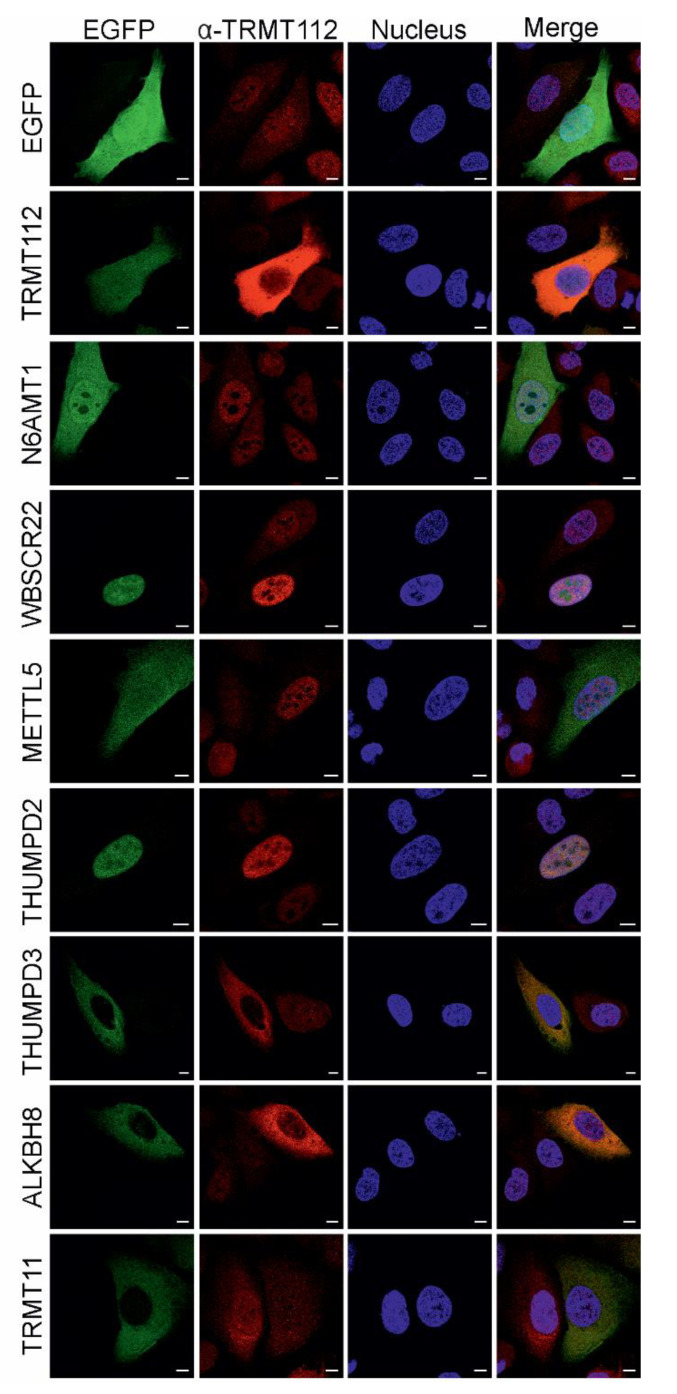
Subcellular localisation of TRMT112 and its partner MTases in U2OS cells. EGFP-fused MTases were expressed in U2OS cells, and their localization, together with endogenous TRMT112 protein, was analysed. The signal of EGFP was revealed through its own fluorescence and TRMT112 with specific monoclonal antibodies to TRMT112, and secondary antibody conjugated with Alexa-568. Cells were counterstained with DAPI for DNA labelling. Image sections were captured with Zeiss LSM710 confocal microscope at 63x magnification. Scale bar 10 µm.

Next, we studied the subcellular localisation of TRMT112 and its partner MTases by overexpressing EGFP-fused MTases in U2OS cells (Figure 2) and then counterstaining the cells with the TRMT112 primary antibody to follow co-localisation. The endogenous TRMT112 protein itself is localised in both the cytoplasm and nucleus in U2OS cells and cells expressing EGFP (Figure 2 and Figure S1). TRMT112-EGFP localisation is similar to the localisation of free EGFP, where the signal is evenly distributed across the cell. EGFP-N6AMT1 and EGFP-METTL5 were detected all over the cell, whereas EGFP-WBSCR22 and EGFP-THUMPD2 displayed a strong nuclear signal. Furthermore, EGFP-WBSCR22 accumulated in the nucleolus, similar to our previous study [9]. EGFP-THUMPD3, EGFP-ALKBH8 and EGFP-TRMT11 all exhibited cytoplasmic localisation. The partner MTases co-localised with the endogenous TRMT112 protein, either in the cytoplasm or nucleus of the cell, and, in several cases, overexpressed MTase determined the localisation of endogenous TRMT112. Our results indicate that TRMT112 accumulates in the nucleus and is not significantly detected in the nucleolus in cells expressing EGFP-WBSCR22 and EGFP-THUMPD2, whereas, in the case of EGFP-ALKBH8 and EGFP-THUMPD3, TRMT112 accumulates in the cytoplasm (Figure 2). The subcellular localisation of endogenous MTases using commercially available antibodies is shown in Figure S1. In some cases, overexpressed proteins are localised differently from endogenous ones. It is worth noting that most of the antibodies used herein are polyclonal and may sometimes show a unspecific pattern.

### 2.3. TRMT112 Stabilises All Seven Methyltransferases in Cells

**Figure 3:**
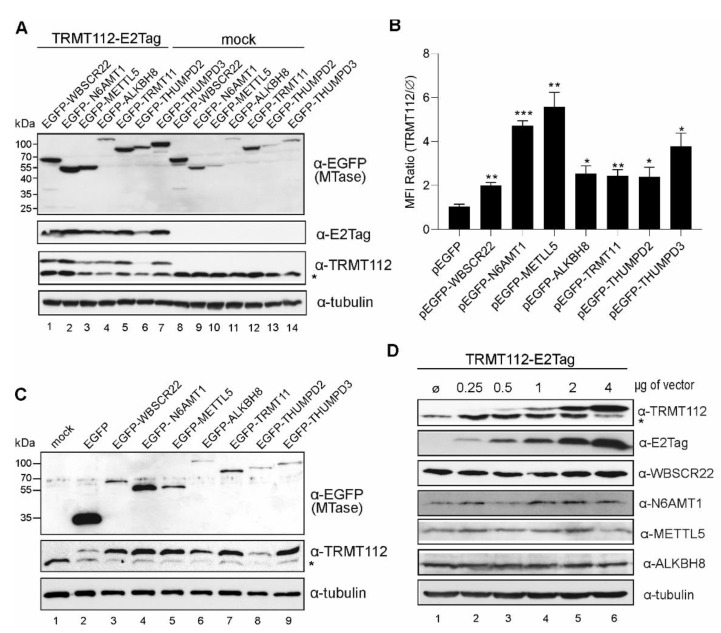
TRMT112 and MTases mutually stabilise each other’s expression in cells. (**A**) TRMT112 stabilises the expression of partner MTases in cells. The EGFP-fused MTases were expressed with and without TRMT112-E2Tag in U2OS cells, and their expression was analysed by Western blot using antibodies against EGFP (to detect MTases), E2Tag (recombinant TRMT112), TRMT112 (both endogenous, marked with asterisk, and recombinant TRMT112, upper band) and β-tubulin (loading control). (**B**) Flow cytometry analysis of the expression level of EGFP-fused proteins after co-expression with TRMT112. Mean fluorescent intensity (MFI) ratios are calculated as relative values of methyltransferase expression with and without TRMT112. EGFP alone was set to 1. Average of three biological replicates are shown. The difference in the sample value compared to control sample value is statistically significant with *p*-value * < 0.05, ** < 0.01 and *** < 0.001. (**C**) TRMT112 protein expression is stabilized by co-expression of its partner MTases. Cells were analysed 48 h after transfection with Western blotting using antibodies against EGFP, TRMT112 and tubulin. Mock-cells without added expression vectors. (**D**) Increasing TRMT112 protein level in cells does not affect the endogenous level of its partner MTases. U2OS cells were transfected with increasing amounts of TRMT112-E2Tag plasmid construct and cells were analysed 48 h later with Western blotting using antibodies against TRMT112, E2Tag, WBSCR22, N6AMT1, METTL5, ALKBH8 and tubulin (50 kDa, loading control).

In our previous works, we noticed that the expression of recombinant WBSCR22 and N6AMT1 proteins, together with TRMT112, stabilised both proteins in cells [9,25]. To study whether this is the case for all partners, we transfected the cells with expression plasmids encoding for EGFP-MTases with (Figure 3A, lanes 1–7) and without (Figure 3A, lanes 8–16) the TRMT112-E2Tag construct, and analysed the cells by Western blot. Co-expression with TRMT112 enhanced the expression level of all EGFP-MTases (Figure 3A, upper panel). Cells were additionally analysed with flow cytometry, where the MFIs (mean fluorescent intensity) of EGFP-MTase proteins were measured, and the activation of MTase expression was calculated as the MFI ratio with and without TRMT112 (Figure 3B). TRMT112 did not affect the expression level of EGFP alone, and this was set as 1. The expression of TRMT112 noticeably increased the amount of all recombinant MTases in the cells, though to a different extent. The biggest effect, which was almost five times, was seen for N6AMT1 and METTL5, and a more modest increase of only 2–3 times was seen for WBSCR22, ALKBH8, TRMT11, THUMPD2 and THUMPD3.

Next, we used two different antibodies to analyse the TRMT112 expression level—the antibody against the epitope tag (E2Tag) recognises the recombinant protein only (Figure 3A), whereas the anti-TRMT112 antibody detects both endogenous and recombinant proteins (Figure 3A,C). Here, we observed that TRMT112 co-expression with MTases not only increased the recombinant MTase expression (Figure 3A) but also increased the level of recombinant TRMT112 itself in the cells (Figure 3C). As shown in Figure 3C middle panel, the expression of TRMT112 was enhanced by co-expression with all MTases except THUMPD2. We also noticed that the endogenous level of TRMT112 was lower when the cells express the recombinant TRMT112 protein (Figure 3C). The same phenomenon was seen in the case of stable TRMT112 cell lines, where the endogenous protein level was decreased in stably expressing U2OS-TRMT112-E2Tag cells (Figure 1A,B).

Next, we wanted to find out whether increasing the TRMT112 levels in cells affects the endogenous levels of its interacting MTases. For this, cells were electroporated with increasing amounts of the TRMT112-E2Tag construct into U2OS cells and analysed 48 h post-transfection. As shown in Figure 3D, the amount of exogenous TRMT112 (as detected by the anti-E2Tag antibody) can be increased, but, at a certain point, the level of endogenous TRMT112 started to drop. At the same time, no major changes were detected in the expression levels of the WBSCR22, N6AMT1, METTL5 and ALKBH8 proteins (Figure 3D).

We suggest that the amount of the TRMT112 protein in cells is strictly regulated and must be on a certain level in order to fulfil its role as a cofactor for MTases. Our results show that recombinant TRMT112 and MTases can mutually regulate each other’s levels in the cell and can exhibit a strong mutual feedback loop when expressed exogenously.

### 2.4. TRMT112 Expression Levels and Stability in Cells Are Strongly Affected by Single Amino Acid Mutations

**Figure 4:**
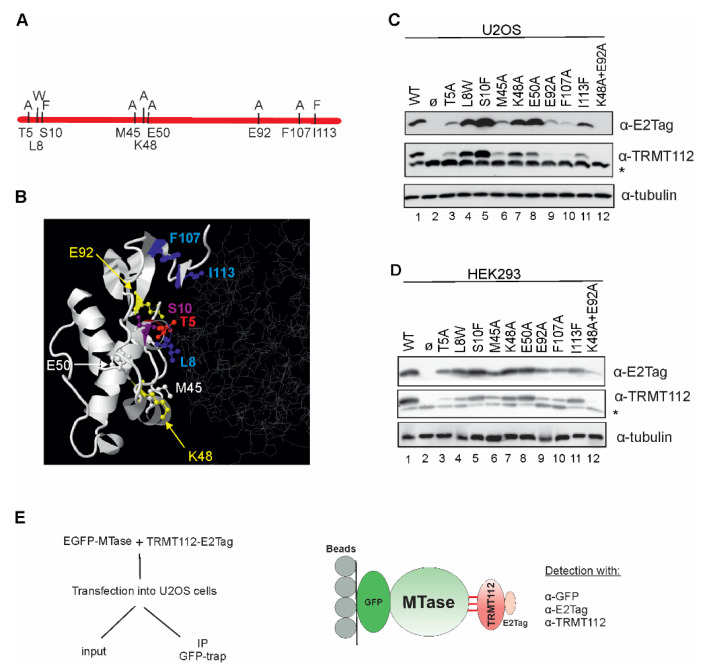
TRMT112 expression and stability is affected by single amino acid mutations. (**A**) Schematic view of point-mutations made in this study. Original amino acids are shown under the line and mutations on the line. (**B**) Localisation of point-mutations on the crystal structure of METTL5-TRMT112 complex (PDB code 6H2U). The structure is visualised with Jmol (http://www.jmol.org/; 08/2021), where TRMT112 is depicted in white ribbon mode and METTL5 in grey wireframe. Mutated amino acids are shown as coloured ball and stick structures. (**C**) U2OS and (**D**) HEK293 cells were transfected with plasmids encoding for E2Tagged WT TRMT112 (line 1) and TRMT112 containing single amino acid mutations (lanes 3–12) and an empty vector (lane 2). Cells were analysed 48 h later using antibodies against E2Tag, TRMT112 and tubulin. Images of results with anti-TRMT112 depict both recombinant (upper band) and endogenous (lower band marked with asterisk *) protein. (**E**) Experimental scheme of the co-immunoprecipitation assay used in this study.

Recent structural analyses have revealed that, in all TRMT112-MTase complexes studied so far, TRMT112 interacts with MTases in a similar way. In the N6AMT1-TRMT112 complex, as well as the METTL5-TRMT112 complex, proteins interact with each other through a large surface (1200 Å and 1160 Å, respectively) characterised by the presence of a large hydrophobic core surrounded by hydrophilic interactions [10,21]. To investigate whether TRMT112 interacts similarly with all of its partners in cells, several amino acids locating on the interface of the TRMT112-MTase complex were mutated. Mutations were chosen based on crystallographic data, as well as an in silico analysis (see Materials and Methods). Amino acids Leu8, Met45 and Ile113 of TRMT112 forming hydrophobic interactions with both N6AMT1 and METTL5 [10,21] were replaced with L8W, M45A and I113F, respectively. Hydrogen-bond forming amino acids Thr5, Ser10 and Lys48 [21] were substituted with T5A, S10F and K48A. In addition, we made mutations E50A, E92A and F107A and the double-mutation K48A + E92A. Replaced amino acids were chosen based on calculations on the effect of mutations using the FoldX program. The mutations and their localisation on the METTL5-TRMT112 complex are shown in Figure 4A,B.

At first, the effect of TRMT112 mutations on its expression level in U2OS cells was studied. Mutations T5A, M45A, E92A and F107A and the double mutation K48A + E92A significantly reduced, but S10F increased the expression level of TRMT112 in U2OS cells (Figure 4C). At the same time, mutations L8W, K48A and E50A did not change the expression level of mutant TRMT112s (Figure 4C). Similar results were obtained from another cell line, HEK293 cells (Figure 4D). As previously shown in Figure 3, TRMT112 protein levels in the cell are tightly regulated in U2OS cells. This phenomenon was also seen in HEK293 cells, where the expression of TRMT112 mutants influenced the endogenous level of the TRMT112 protein (Figure 4D, middle image).

### 2.5. TRMT112 Mutants Reveal Similarities, but Also Differences, While Interacting with MTases in the Cells

**Figure 5:**
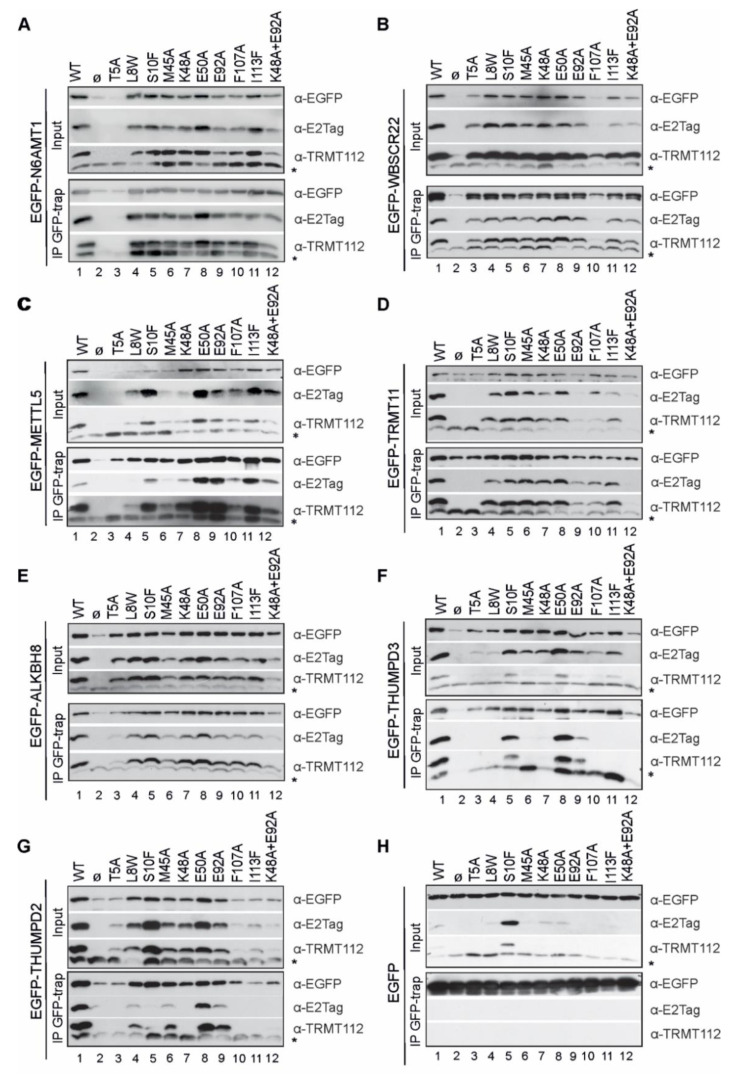
TRMT112 single amino acid mutant interactions with MTases confirm similarities, but also reveal differences in the way TRMT112 interacts with its partners. U2OS cells were transfected with plasmids encoding for EGFP-tagged N6AMT1 (**A**), WBSCR22 (**B**), METTL5 (**C**), TRMT11 (**D**), ALKBH8 (**E**), THUMPD3 (**F**), THUMPD2 (**G**) and EGFP (**H**), along with plasmids encoding for wt or mutated E2Tag-containing TRMT112. Immunoprecipitation was performed with GFP-Trap beads 48 h later using EGFP-tagged MTase as a bait, and samples were subsequently analysed using Western blot. Upper three images of A-H panel represent input of total soluble cell lysate, whereas lower three images represent immunoprecipitated material. Both sections were analysed using antibodies against EGFP, E2Tag and TRMT112. Images of results with anti-TRMT112 depict both recombinant (upper band) and endogenous (lower band marked with asterisk *) protein.

Next, we transfected the cells with two expression vectors, encoding for EGFP-MTase and E2Tag-TRMT112 or its mutant, and performed an immunoprecipitation assay with GFP-Trap beads using EGFP-MTase as a bait, as depicted in Figure 4E. In Figure 5, input sections depict the soluble fraction of samples, whereas IP GFP-trap panels show immunoprecipitated proteins. EGFP-MTase proteins were able to interact with both recombinant and endogenous TRMT112 proteins and immunoprecipitated them from cells. Again, a strong stabilisation effect obtained with co-expression of MTases and TRMT112 in cells was observed (Figure 5; lanes 1, 2). Results reveal that TRMT112 mutant-MTase interactions have numerous similarities, along with several differences. Based on the crystallographic data, amino acids Leu8, Met45 and Ile113 of TRMT112 are directly involved in hydrophobic interactions with both N6AMT1 and METTL5 [10,21]. In our experiment, mutations L8W and M45A did not affect the TRMT112 interaction with N6AMT1, WBSCR22 and TRMT11 (Figure 5A,B,D; lanes 4, 6), but, in both cases, disturbed the TRMT112 interaction with METTL5 and THUMPD3 (Figure 5B,F; lanes 4, 6), whereas the interaction with ALKBH8 was impacted only by M45A (Figure 5E; lanes 4, 6). I113F had no effect on either of them (Figure 5A–E; lanes 11). All three mutations L8W, M45A and I113F disturbed the THUMPD3-TRMT112 and THUMPD2-TRMT112 interactions (Figure 5F,G; lanes 11). The replacement of the phenylalanine in position 107 with alanine (F107A) influenced the TRMT112 interaction with WBSCR22, THUMPD2 and THUMPD3, had a less noticeable effect on TRMT11, and did not influence interactions with N6AMT1, ALKBH8 and METTL5 (Figure 5; lane 10).

Hydrogen-bond-forming amino acid Thr5 was replaced with alanine, and this mutation strongly affected the stability of the TRMT112 protein in cells (Figure 4 and Figure 5) and appeared to disturb the interaction with TRMT112 in almost all cases, especially for N6AMT1, METTL5, TRMT11, THUMPD2 and THUMPD3. In contrast, the replacement of the other polar amino acid Ser10 with phenylalanine (S10F) (Figure 5; lane 5) enhanced the expression of the TRMT112 protein in the cells (Figure 3C); furthermore, S10F disturbed the MTase-TRMT112 interaction only in the case of THUMPD2. The mutation K48A affected the TRMT112 interaction with THUMPD2 and THUMPD3, but not in any of the other cases (Figure 5; lanes 7). E92A did not show any effect, or had only a very mild effect on N6AMT1, WBSCR22, METTL5 and ALKBH8, but disturbed the THUMPD2 and THUMPD3 interaction (Figure 5; lanes 9). Furthermore, E92A, together with K48A, affected the interaction with TRMT11, THUMPD2 and THUMPD3 (Figure 5D,F,G; lanes 12). E50A did not localise on the interface surface of the TRMT112-MTase interaction and did not have an effect in any case (Figure 5; lanes 8).

In summary, the TRMT112-interacting MTases can be conditionally divided into two groups: the first group consists of N6AMT1, METTL5, WBSCR22, ALKBH8 and TRMT11, and the second group consists of THUMPD2 and THUMPD3. In the case of the first group, the defective TRMT112 mutant proteins almost always affected the partner MTase stability when co-expressed together, so that the effect was already seen in the input (as detected by E2Tag antibodies). For example, there is no signal either in the input or IP fraction for T5A for N6AMT1, F107A for WBSCR22, etc. The second group includes, in addition to the above, TRMT112 mutants that are expressed at near wt level, like S10F and K48A for THUMPD2 and M45A, K48A and I113F for THUMPD3, but we were unable to immunoprecipitate them using the GFP-fused MTase. This suggests that the THUMPD2 and THUMPD3 interaction with TRMT112 is more complex and is affected by some additional proteins or biomolecules in cells.

## 3. Discussion

TRMT112 has been shown to interact with several MTases while enhancing their stability and catalytic activity. Using a SILAC screen, we pulled down seven MTases—already known partners N6AMT1, WBSCR22, METTL5 and ALKBH8, and two novel MTases THUMPD2 and THUMPD3 specific to mammalian cells, as well as TRMT11, which, so far, has only been studied in yeast. Thus, together with already known proteins, TRMT112 is a partner for at least seven different MTases in mammalian cells.

Most of the knowledge about eukaryotic TRMT112 and its orthologues comes from studies in yeast, whereas mammalian cells have a markedly more complex internal environment with thousands of different biomolecules, where their protein function is dependent on epigenetic regulation, interactions with other molecules and the availability of substrates, as well as compartmentalisation. We show that the recombinant TRMT112 stabilises all seven MTases when expressed together in mammalian cells. This is consistent with previous works, where the metabolic stabilisation of WBSCR22 and METTL5 by TRMT112 was shown [9,10,12]. We also observed a strong mutual feedback loop—recombinant MTases, except THUMPD2, stabilise the TRMT112 protein in cells. In addition, we observed a fluctuation in endogenous TRMT112 levels—sometimes the endogenous TRMT112 level increased, and sometimes it decreased. As the endogenous TRMT112 level in stable cell lines always decreased, the increased TRMT112 amount we observe in TRMT11-MTase co-expression studies is most likely temporary. Our data suggest that there is a dynamic TRMT112-MTase network in the cells, where the amount of each MTase depends on the amount of free TRMT112 available and vice versa. More generally, the amount of both partners, TRMT112 and MTase, depends on the amount of free partner proteins available to form a heterodimer. The unbound TRMT112, and also some MTases, such as WBSCR22 [9], are likely to be degraded. In this model, the amount of TRMT112 is a critical factor that determines the number of enzymatically active MTases in cells. However, when gradually increasing only the amount of exogenous TRMT112 in the cell, the endogenous level of N6AMT1, WBSCR22, METTL5 and ALKBH8 remained mostly at the same level (Figure 3D). This shows that the cell itself cannot increase MTase expression in response to an increased TRMT112 amount in the cell.

**Figure 6:**
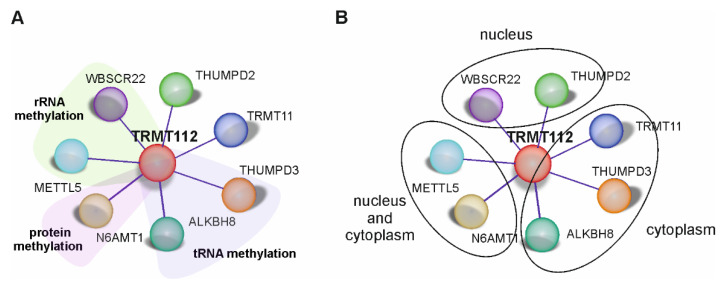
TRMT112 interactome. MTases interacting directly with TRMT112 are shown together with their known enzymatic activities (**A**) and cellular localisation (**B**).

Mammalian TRMT112 is localised to both the nucleus and cytoplasm of the cell, where it interacts and forms complexes with partner MTases: with WBSCR22 and THUMPD2 in the nucleus, with N6AMT1 and METTL5 in the nucleus and cytoplasm and with THUMPD3, ALKBH8 and TRMT11 mainly in the cytoplasm (Figure 6). Our data are consistent with that of Garcia et al., where the Myc-tagged TRMT112, WBSCR22, N6AMT1, METTL5, ALKBH8 and TRMT11 overexpressed in Borna-disease-virus-1-infected human oligodendriocytes displayed a similar pattern [35]. During immunofluorescence studies, it was observed that, when TRMT112 partner MTases were overexpressed in cells, endogenous TRMT112 showed an increased signal and changed its cellular localisation to follow the localisation of its MTase partner. Results indicate that WBSCR22 and THUMPD2 overexpression increases the amount of TRMT112 in the nucleus while reducing its cytoplasmic expression, but THUMPD3 and ALKBH8 overexpression increases the TRMT112 amount in the cytoplasm while reducing its signal in the nucleus. A re-localisation of recombinant TRMT112 when it is overexpressed simultaneously with exogenous WBSCR22 has also been detected in previous research. Õunap et al. have shown that mCherry-tagged TRMT112 is detected all over the cell, but, when co-expressed with EGFP-tagged WBSCR22, it relocates to the nucleus. The exogenously expressed MTase presumably forms a heterodimer with free TRMT112, which is otherwise probably degraded and relocalises it to its cell compartment. Currently, it is not known how much free TRMT112 protein there is in the cells, and how much of it is in a complex with other proteins. It is also not known how stable TRMT112-MTase heterodimers are in the cells and whether they disassociate once formed. TRMT112 covers a large hydrophobic cluster of the partner protein and partially protects it from the external environment, which is probably the major determinant of the stability of heterodimers [10,21]. It is doubtful that, once formed, heterodimers dissociate, thus allowing for TRMT112 to interact with another partner; it is far more likely that already formed TRMT112-MTase heterodimers are stable in cells. However, we cannot rule out that TRMT112-MTase complexes in the cell are dynamic, thus allowing for TRMT112 to interact with another MTase if a partner with a stronger binding potential is detected. Nonetheless, it is not possible to confirm any of these hypotheses at this point in our research.

The crystal structure analyses of TRMT112 and MTase complexes studied so far have revealed that TRMT112 interacts with its partners in a similar way. All TRMT112 partners bind to the same region of the MTase domain. TRMT112 does not bind closely to the active site on the MTases, and it appears that TRMT112 is not directly involved in substrate binding and recognition, as it does not have a direct effect on the chemical structure and binding properties of the MTase [21]. Nonetheless, the crystal structures of human TRMT112 are available only with the N6AMT1 and METTL5 complex; therefore, it cannot be ruled out that some unknown differences exist. The data obtained from this study have revealed that certain mutations on different parts of the TRMT112 binding surface affect its MTase partners in different ways, resulting in unaffected, reduced or even no binding of TRMT112 at all. Therefore, it can be suggested that, although TRMT112 interacts with its partners in a similar way, there are subtle but nevertheless important differences.

The biological activities of TRMT112 are thought to be the result of the impaired metabolic activities of partner MTases, as TRMT112 binding stabilises MTases while also enhancing the substrate binding [8,30]. Indeed, the down-regulation of TRMT112 significantly reduced the WBSCR22 protein levels in cells, and complementation with the recombinant protein expressed from the transfected plasmid restored it [9,10,12]. Growth inhibition was observed after the down-regulation of WBSCR22 itself, and can be explained with defects in rRNA processing and the maturation of small ribosomal subunits [18]. Intriguingly, the MTase activity of WBSCR22 is not required for its activity in ribosome synthesis [12], suggesting that the WBSCR22 protein itself has additional activities than MTase, and the role of TRMT112 may be just to stabilise the WBSCR22 protein. METTL5 is also metabolically stabilised by TRMT112; however, the knock-down of METTL5 with siRNAs does not affect the cell growth [10]. At the same time, the bi-allelic variants in ***METTL5*** resulting in truncated METTL5 with a missing C-terminus causes autosomal-recessive intellectual disability and microcephaly, highlighting its essential role in brain development and cognitive function [36]. An even more diverse partner is N6AMT1, which is metabolically stable also without TRMT112 [25]. In the current work, the N6AMT1-TRMT112 interaction was less affected by TRMT112 mutations than the interaction with other MTases. TRMT112-N6AMT1 heterodimers possess MTase activities that are required for protein; specifically, the glutamine of the motif GGQ in ETF1 and histone H4 lysine, methylation [8,20,23]. Meanwhile, their involvement in DNA N6-adenine methylation is still debatable [21,22]. TRMT112 also forms a complex with TRMT11, which has not yet been characterised in human cells. The TRMT11 homologue in yeast Trm11 forms a complex with the TRMT112 homologue Trm112, where it is involved in tRNA methylation [15]. Human TRMT11 is involved in carcinogenesis as part of a fusion gene with GRIK2, which is found in many cancer types [37,38,39]. Currently, it is predicted that TRMT11 has a tRNA MTase activity in human cells [40]; nonetheless, more studies are needed to elucidate the functions of TRMT11. Finally, TRMT112 partners THUMPD2 and THUMPD3 are especially interesting, as TRMT112 mutations affect them the most, suggesting that these proteins interact with TRMT112 slightly differently than the other MTases.

In summary, human TRMT112 interacts with at least seven different MTases, thus emphasizing its importance as a central “hub” protein regulating the activity of more than one MTase. TRMT112-MTase heterodimers are involved in various biological processes, such as ribosome biogenesis, protein translation, cell proliferation, the functioning of mitochondria, etc., through their activity in rRNA, tRNA and protein methylation. There are still a lot of open questions; for example, how exactly the TRMT112 activity in cells is regulated and whether it has additional functions on its own that are independent of its role as a co-activator of MTases. RNA methylation has recently received great attention and has experienced a rapid increase in its research development. Further extensive investigations and new discoveries relating to its regulation and function in organisms will provide a comprehensive understanding of the biology of methylation and the role of MTases in it. There is a lot of evidence that TRMT112 partners are involved in various human pathologies; therefore, a further understanding of TRMT112-MTase heterodimer functions in normal, as well as pathological, processes could lead to them being used as drug targets in future.

## 4. Materials and Methods

### 4.1. Plasmids

Plasmids encoding for the open reading frames of TRMT112, N6AMT1 and WBSCR22 are described in [9,18,25]. Open reading frames for THUMPD2 (Q9BTF0), THUMPD3 (P97770), TRMT11 (Q7Z4G4), METTL5 (Q9NRN9) and ALKBH8 (Q96BT7) were amplified from the cDNA obtained from U2OS cells and cloned into pQM (Icosagen Ltd., Tartu, Estonia) and pEGFP-C1 plasmids, respectively, in a similar way. In all cases, EGFP and E2Tag are located in the N-terminus of a MTase. TRMT112-EGFP and pQM-TRMT112 plasmids were used to introduce mutations into TRMT112 sequence. Mutagenesis was carried out using the polymerase incomplete primer extension method [41] with primers listed in Supplementary Table S1. All of the coding regions and mutations were confirmed by Sanger sequencing.

### 4.2. Cell Culture and Transfections

Human osteosarcoma (U2OS) and human embryonic kidney cells (HEK293), obtained from ATCC (American Type Culture Collection; Manassas, VA, USA), were grown in Iscove’s Modified Dulbecco’s Media (IMDM) supplemented with 10% fetal calf serum (FCS) (Gibco, Thermo Fisher Scientific, Waltham. MA, USA), 100 U/mL penicillin and 100 µg/mL streptomycin. Cells were incubated at 37 °C in 5% CO_2_ environment.

For protein expression analysis, 250 µL of U2OS or HEK293 cell suspension (2 × 10^6^) was mixed with salmon sperm carrier DNA and 1 µg of EGFP or pQM plasmids encoding for proteins of interest in 4 mm cuvettes and transfected by electroporation using Bio-Rad GenePulser Xcell (settings 200 V, 975 µF) (Hercules, CA, USA). The cells were suspended in culture media, plated to 100 mm cell culture dishes and analysed 24 h and 48 h after transfection by Western blot analysis.

Stable cell lines expressing TRMT112-E2Tag protein in U2OS cells were generated as described in [9]. Shortly, pQM-TRMT112 and pBabePuro plasmids were linearised, ligated to form dimers, and transfected to U2OS cells. Twenty-four hours after transfection, puromycin at final concentration 5 μg/mL was added to the media. In pQM-TRMT112, the strong CMV promoter was replaced with HSP promoter for moderate expression level of TRMT112 protein. Two weeks after transfection, colonies were selected and the expression of TRMT112 protein was analysed by immunoblotting. For SILAC analysis, U2OS-TRMT112-E2Tag cells were grown in SILAC DMEM supplemented with 10% dialysed fetal bovine serum, heavy arginine (0.133 mM, CNLM-539) and heavy lysine (0.266 mM; CNLM-291) (Cambridge Isotope Laboratories Inc., Tewksbury, MA, USA) for 7 days.

### 4.3. Immunoprecipitation

U2OS cells were transfected, seeded on 100 mm cell culture dishes and collected 48 h after transfection in 500 μL of IP lysis buffer (150 mM NaCl; 20 mM Hepes (pH = 7.2), 100 mM K-acetate, 2mM MgCl_2_, 0.1% Tween-20, 1% Triton X-100, H_2_O and protease inhibitor (Complete ULTRA Tablets, Roche). Then, 50 μL was taken for input control and 450 μL was used for immunoprecipitation assay with GFP-Trap magnetic beads (ChromoTek GmbH, Munich, Germany) according to the manufacturer’s protocol.

### 4.4. Immunoblotting

Live cells in PBS buffer, cell lysates in IP buffer or GFP-Trap magnetic beads with attached proteins were reduced with DTT in 2x Laemli buffer and denatured with 100 °C heat. Ten µL of protein lysates were separated electrophoretically using 10% SDS-PAGE gel and blotted onto a PVDF membrane using Trans-Blot SD Semi-Dry Transfer Cell (BioRad). The proteins were detected using anti-E2-Tag antibody 5E11 (1:10,000, Icosagen, Tartu, Estonia), anti-α-tubulin (1:10,000, Sigma-Aldrich, St. Louis, MO, USA), anti-TRMT112 (1:1000, sc-398481, Santa Cruz Biotechnology, Dallas, TX, USA), anti-WBSCR22 (1:1000, sc-376714; Santa Cruz Biotechnology, Dallas, TX, USA), anti-METTL5 antibody (1:1000, HPA038223, Atlas antibodies, Bromma, Sweden), anti-ALKBH8 (1:500, HPA061514, Atlas antibodies, Bromma, Sweden), anti-N6AMT1 (1:500, HPA059242, Atlas antibodies, Bromma, Sweden) anti-TRMT112 (1:1000, HPA04006, Sigma-Aldrich, Saint Louis, MO, USA), anti-TRMT112 (1:3000, HPA039901, Atlas antibodies, Bromma, Sweden) and EGFP (1:10,000, Institute of Technology; University of Tartu, Tartu, Estonia). Goat anti-rabbit (1 mg/mL, LabAS) and goat anti-mouse (1 mg/mL, LabAS) antibodies were used as secondary antibodies. Detection was performed using an ECL detection kit (GE Healthcare, Chicago, IL, USA) following the manufacturer’s instructions.

### 4.5. Immunofluorescence Microscopy

U2OS cells were transfected and seeded on coverslips in 24-well plates. Forty-eight hours post transfection, the cells were washed with PBS, fixed with 4% paraformaldehyde for 10 min and permeabilised with 0.2% Triton-X-100 for 10 min on ice. Samples were blocked with 3% BSA/PBS solution for 1 h at RT and stained with anti-TRMT112 primary antibody (1:50, sc-398481, Santa Cruz Biotechnology, Dallas, TX, USA) diluted in 3% BSA/PBS solution, followed by 3 washes with PBS and incubation with secondary anti-mouse antibody conjugated to Alexa Fluor 568 (1:1000, Invitrogen, Carlsbad, CA, USA) diluted in 3% BSA/PBS solution. Nuclei were counterstained with DAPI and EGFP were detected using their fluorescence. The samples were analysed using a Zeiss LSM 710 confocal microscope and Zen blue 3.1 software (Carl Zeiss AG, Oberkochen, Germany).

### 4.6. Flow Cytometry

U2OS cells were transfected and collected 48 h later, washed with PBS and analysed with Attune NxT Flow Cytometer (Thermo Fischer Scientific, Waltham, MA, USA) according to manufacturer’s instructions. Analysis was performed with FlowJo (Beckton Dickinson, Franklin Lakes, NJ, USA) software.

### 4.7. Proteomic Analysis

Proteomic analysis was performed according to the protocol described in [9].

### 4.8. In Silico Analysis for Predicting Mutations Defective on Specific Trmt112-MTase Interactions

The FoldX program was used to calculate the effect of mutations on stability of Trmt112:MTase complex and for Trmt112 monomers (extracted from complex structure). For METTL5:Trmt112 complex, we used PDB structures 6H2U and 6H2V; the latest contains two heterodimeric complexes and, respectively, we used two separate heterodimers for calculations. First contains chains A and B and second contains chains C and D. For N6AMT1:Trmt112 complex, we used PDB structures 6K0X, 6H1D, 6H1E and 6PED.

FoldX4 was used to evaluate the effect of alanine scanning mutagenesis of methyl transferases and Trmt112 on MTase:TRMT112 complex formation. ’AlaScan’ command was applied to energy-minimised structures (with the same temperature and ionic strength parameters). For each position, ddG(complex)-ddG(TRMT112 monomer) was calculated to evaluate the specific effect on heterodimerisation. This approach does not find any mutation having destabilizing effect only on specific complex. Next, FoldX command ‘AnalyseComplex’ with default parameters was used to identify Trmt112 amino acids close to the interaction interface on energy-minimised structures. All found amino acids where in silico mutated into every single amino acid (FoldX ‘PositionScan’ command, temperature and ionic strength parameters as before), and data were analysed to find mutations with selective effect on complex stability. General (but not strict) rules for selecting mutations: (a) destabilizing effect of mutations (ddGcomplex-ddGTrmt112 monomer) to at least one interaction of at least 2 kcal/mol, and leaving at least one interaction unchanged; (b) mutation effect (ddG) on free Trmt112 should be minimal (between 1 and −1 kcal/mol). For specific distortion interaction between N6AMT1 and Trmt112, we did not find mutations fulfilling above criteria. We selected the mutations closest to the criteria.

## Data Availability

The data and materials presented in this study are available on request from the corresponding author.

## Supplementary Materials

The following are available online at https://www.mdpi.com/article/10.3390/ijms222413593/s1.
