# Supplementary material for: Human TRMT112-Methyltransferase Network Consists of Seven Partners Interacting with a Common Co-Factor
Source: Int J Mol Sci. 2021 Dec 18;22(24):13593. doi: 10.3390/ijms222413593 (PMC8708615; doi:10.3390/ijms222413593)
Supplement: Supplementary file 1 [file ijms-22-13593-s001.zip › ijms-1438531-supplementary.pdf]

Table S1. Primer sequences used for TRMT112 mutagenesis

Primer name	Primer sequence 5'>3
Thr5Ala_1	CTTGCCCACAATCTGCTGAGCTCGCATGTG
Thr5Ala_2	GTGGGCAAGCAGTTTCATAAGAGATCCC
Leu8Trp_1	AATTGGCTGAGCTCGCATGTGCGGGGGGTG
Leu8Trp_2	CAGCCAATTGTGGGTAAGCAGTTTCATAAG
Ser10Phe_1	CTGTTCTCGCATGTGCGGGGGGTGGGG
Ser10Phe_2	CGAGAACAGCAGATTGTGGGTAAGCAG
Met45Ala_1	CGTGCGATACCTAAAGTGGAGTGGTCGG
Met45Ala_2	TATCGCACGCGCCACGAAGTTGGGGTTG
Lys48Ala_1	CCTGCAGTGGAGTGGTCGGCGTTCCTGG
Lys48Ala_2	CACTGCAGGTATCATACGCGCCACGAAG
Glu50Ala_1	GTGGCGTGGTCGGCGTTCCTGGAGGCGG
Glu50Ala_2	CCACGCCACTTTAGGTATCATACGCGCC
Glu92Ala_1	GTGGCAGTGATAGAGGGCACCCTGCAGTGC
Glu92Ala_2	CACTGCCACCTCCAGCAGCAGGTGGTGCATG
Phe107Ala_1	ATGGCCCCCATCAGCCGCGGGATCCCCAAC
Phe107Ala_2	GGGGGCCATACGTCCAGATTCCGGGCACTG
Ile113Phe_1	GGGTTCCCCAACATGCTGCTGAGTGAAGAGG
Ile113Phe_2	GGGGAACCCGCGGCTGATGGGGAACATACGT
